# Interpersonal stress, epigenetic indices of inflammation, and depressive symptoms: Longitudinal associations from adolescence to young adulthood

**DOI:** 10.1016/j.ynstr.2026.100832

**Published:** 2026-06-22

**Authors:** Stefanos Mastrotheodoros, Winni Schalkwijk, Michelle de Groot, Sinan Gülöksüz, Stefanie A. Nelemans, Jim van Os, Bart P.F. Rutten, Wim Meeus, Marco P. Boks, Susan Branje

**Affiliations:** a Section of Youth and Family, Dept. of Education and Pedagogy, Utrecht University, Utrecht, the Netherlands; b Department of Psychology, University of Crete, Crete, Greece; c Department of Psychiatry, Brain Center, University Medical Center Utrecht, Utrecht, the Netherlands; d Department of Psychiatry and Neuropsychology, Mental Health and Neuroscience Research Institute, Maastricht University Medical Center, Maastricht, the Netherlands; e Department of Psychiatry, University of British Columbia, Vancouver, BC, Canada; f Institute of Mental Health, University of British Columbia, Vancouver, BC, Canada; g Northern Medical Program, Division of Medical Sciences, University of Northern British Columbia, Prince George, BC, Canada; h Department of Psychiatry, Yale University School of Medicine, New Haven, CT, USA; i Department of Human Genetics, Radboud University Medical Center, Nijmegen, the Netherlands; j Department of Psychiatry, Amsterdam UMC, Amsterdam, the Netherlands; k Dimence Institute for Specialized Mental Health Care, Deventer, the Netherlands

**Keywords:** Interpersonal stress, Negative parenting, Peer victimization, Inflammation, Epigenetics, DNA methylation, Latent congruence model

## Abstract

**Background:**

Adolescent interpersonal stress increases depression risk, and chronic low-grade inflammation may act as a potential underlying biological mechanism.

**Methods:**

We explored longitudinal associations between interpersonal stress, inflammation, and depressive symptoms from adolescence to young adulthood, using DNA methylation (DNAm) indices of C-reactive protein (CRP) from saliva. Data were collected from *N* = 434 adolescents (RADAR-Y study, 56.4% male) and analyzed in preregistered structural equation models. Interpersonal stress was estimated from repeated measures of parent-adolescent conflict, parental psychological control, parental criticism, and peer victimization.

**Results:**

Interpersonal stress was not associated with DNAm indices of CRP at age 17 or age 25, nor with depressive symptoms at age 27. In exploratory analyses examining parent versus peer-related stress separately, higher negative parenting–but not peer victimization–was nominally associated with one out of three DNAm indices of CRP at age 17 (Hillary index, *β* = .15, *p* = .035), with no association at age 25.

**Conclusion:**

This study did not show an association of either interpersonal stress or inflammation with later depressive symptoms and thereby could not identify inflammation as a potential mediator. Our findings tentatively suggest timing-dependent differential effects of family versus peer-related stress on epigenetic indices of inflammation, which may be further explored in future studies.

## Highlights

Adolescent interpersonal stress increases the risk for depressive symptoms, but underlying biological mechanisms are unclear.
Low-grade chronic inflammation may mediate this relation and can be measured using a DNA methylation index of CRP.
Composite interpersonal stress in adolescence was unrelated to salivary DNAm indices of CRP &amp; depressive symptoms in adults.
Negative parent-adolescent relationship quality—but not peer victimization—was related to higher salivary DNAm-CRP at age 17.

## 1 Introduction

### 1.1 Inflammation as a potential mediator between interpersonal stress and depression

Stress can arise in parent-child and peer relationships, for example in the form of conflict, criticism, or victimization, with important consequences for youth's current and future adjustment and mental health (Beach et al., 2017; Mastrotheodoros et al., 2023; Moore et al., 2017). A potential mechanism linking interpersonal stress to mental health is aberrant immune system activation. While acute stressors trigger appropriate immune responses, chronic stress can disrupt negative feedback interactions between stress hormones and immune cells, impairing fine-tuned co-regulation of both systems (Beurel et al., 2020; Cohen et al., 2012; Dhabhar, 2014). This may result in a state of chronic low-grade inflammation—a steady and persistent immune system activation even in the absence of apparent threats, negatively impacting both physical and mental health (D. Furman et al., 2019).

Several studies link interpersonal stress to inflammation. For instance, harsh and low-warmth parenting during early adolescence predict increased levels of several inflammatory markers in late adolescence (Byrne et al., 2017) and adulthood (Beach et al., 2017; O'Brien et al., 2023). Similarly, experiences of peer victimization during childhood and adolescence are prospectively associated with inflammatory markers (Chen et al., 2023; Scott and Manczak, 2021), including C-Reactive Protein (CRP; Baldwin et al., 2018; Copeland et al., 2014; Iob et al., 2022; Takizawa et al., 2015), Tumor Necrosis Factor-a, and Interleukin-6 (Chen et al., 2023). In fact, childhood victimization might be among the strongest interpersonal predictors of increased CRP in adulthood (Iob et al., 2022). Notably, *repeated stress* might be particularly burdensome. The *number of measurement timepoints* in which participants reported bullying incidents was associated with higher levels of CRP (Copeland et al., 2014).

In turn, chronic inflammation may increase risk of depressive symptoms. Studies report elevated levels of inflammatory markers in clinical depression as compared to controls (Chiang et al., 2019a; Chiang et al., 2019b; Haapakoski et al., 2015). Moreover, longitudinal studies indicate that higher blood levels of inflammatory markers are prospectively linked with higher depressive symptoms in youth (Toenders et al., 2022; Valkanova et al., 2013). Importantly, the association between inflammation and depressive symptoms appears to be bidirectional (Beurel et al., 2020), as depressive symptoms also predicted higher CRP and IL-6 levels (Toenders et al., 2022).

Overall, several studies have shown associations between interpersonal stress and inflammation or inflammation and depressive symptoms, giving rise to the hypothesis that inflammation may mediate the stress–depression link. Given that adolescence is a formative period sensitive to the negative effects of stress, it is important to further examine how persistent stress experienced throughout adolescence might impact inflammatory processes. Understanding inflammation as a potentially modifiable mechanism could ultimately give rise to novel prevention strategies to reduce the negative effects of stress on depressive symptoms in youth.

### 1.2 An epigenetic perspective

Research on the causes and consequences of chronic inflammation is hampered by the high short-term variability of systemic immune markers. A potential solution to this challenge comes from recent studies showing that chronic systemic inflammation is well reflected in the epigenome (Hillary et al., 2024; Wielscher et al., 2022). Specifically, DNA methylation (DNAm) can statistically explain up to 50% of blood levels of CRP (Hillary et al., 2024). This illustrates the inflammation biomarker potential of DNAm, which may result from both direct and indirect factors linking systemic inflammation to the blood DNA methylome, including genetic and environmental influences. Large population epigenome-wide association studies have reliably identified epigenetic indices of CRP which reflect chronic low-grade inflammation (Verschoor et al., 2023) and have previously been applied in studies on depression (Edmondson-Stait et al., 2022; Green et al., 2021; Verschoor et al., 2021). Compared to serum levels of inflammatory markers, DNAm indices showed higher test-retest reliability (Stevenson et al., 2020) and stronger associations with brain areas correlated with depression (Green et al., 2021).

Moreover, DNAm indices of CRP have been validated for studying inflammation in youth (Barker et al., 2018; Raffington et al., 2023) and in saliva samples (Conole et al., 2023; Raffington et al., 2023). Such empirical support is not yet available for epigenetic indices of other inflammatory proteins than CRP (Schalkwijk et al., 2026). Notably, the study by Barker et al. (2018) indicated that the effects of DNAm indices of CRP on later mental health related to the temporal stability of these indices. Therefore, repeated assessment of DNAm might reveal patterns that could not be found otherwise.

### 1.3 Current study

**Fig. 1:**
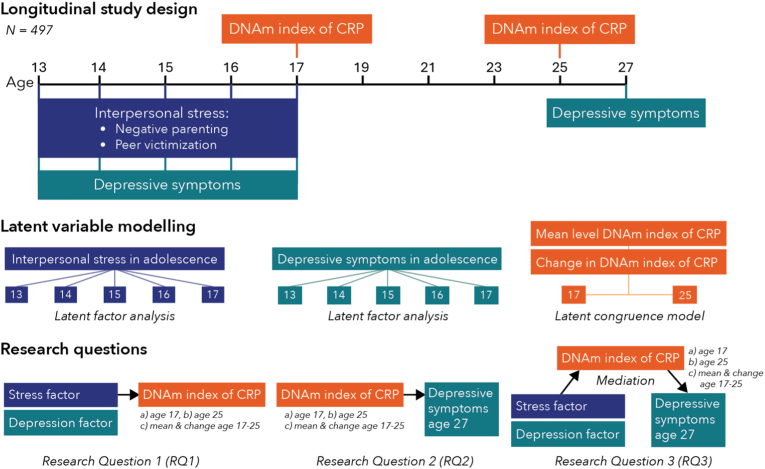
**Study design, analytical approach and research questions (RQs).** RQ1 addresses the effects of interpersonal stress (and depressive symptoms) during adolescence on DNAm indices of CRP. RQ2 addresses the effects of the DNAm indices of CRP on depressive symptoms at age 27. RQ3 addresses the mediating role of DNAm indices of CRP in the link between interpersonal stress during adolescence and depressive symptoms at age 27. In all models, four constructs of DNAm CRP are included, corresponding to sub-questions a (age 17 DNAm), b (age 25 DNAm), c (average DNAm), and d (within-person change in DNAm).

In this study, we investigated prospective associations between interpersonal stress during adolescence (ages 13 to 17), DNA methylation (DNAm) indices of CRP from saliva (ages 17 and 25), and depressive symptoms in young adulthood (age 27). See Fig. 1 for an overview of the study. We aimed to extend prior knowledge in three ways. First, we captured cumulative stress from negative parenting and peer victimization during adolescence—a critical developmental period. Second, we assessed DNAm indices of CRP both at age 17 and age 25 to examine the measurement *timing*, and *within-person changes* in inflammation. Third, we tested the bidirectional associations of depressive symptoms and inflammation, as well as the potential mediating role of inflammation in the stress-depression link. Our pre-registered research questions and hypotheses can be found on the OSF portal (https://osf.io/t2cp4/overview?view_only=8db91c0cb9f44a15915dc49c7e80d990).

## 2 Methods

### 2.1 Participants

Data for this study was collected in the ongoing longitudinal Research on Adolescent Development And Relationships-Young study Branje and Meeus (2018). The original sample consisted of 497 adolescents (*M*_*age*_ = 13.03, *SD* = 0.47 at baseline, 56.4% male) who were mostly native Dutch (94.8%) and lived with both parents (85.2%). The current study used data from the first 11 measurement timepoints. From early to late adolescence (age 13-17), study participants took part in annual assessments, which included questionnaires on parenting, peer victimization, and depressive symptoms. From late adolescence onwards, data collection took place every two years. Saliva samples for DNA extraction were provided by a subsample of *N* = 420 participants in late adolescence (*M*_*age*_=17.03), and again by *N* = 247 participants in young adulthood (*M*_*age*_ = 25.8). Finally, two years later (*M*_*age*_ ≈ 27.8), participants reported their depressive symptoms. To optimize power and given that analyses would apply robust methods to handle missing data (see Analytic Plan), we included the 434 youth who provided saliva data at least once.

### 2.2 Measures

#### 2.2.1 Interpersonal stress

Self-report questionnaires were used to assess negative parenting—specifically psychological control, parent-adolescent negative interactions, and expressed emotion—alongside peer victimization as indicators of interpersonal stress. All four variables were assessed annually from early to late adolescence (13 to 17 years).

Psychological control was assessed with the Psychological Control Scale (Barber, 1996), consisting of eight items, addressed on a 5-point Likert scale from 1 (*Does not describe my mother/father at all*) to 5 (*Describes my mother/father very well*). Adolescents addressed the items with respect to mother and father separately, and a mean score was computed for each measurement timepoint, such that higher scores indicate higher parental psychological control. An example item is “My mother/father often interrupts me”. Cronbach's α ranged from .75 to .87 across timepoints.

Parent-adolescent conflict intensity was assessed with the Negative Interactions subscale of the Network of Relationships Inventory-short form (De Goede et al., 2009; W. Furman and Buhrmester, 1985), which consists of six items addressed on a 5-point Likert scale, ranging from 1 (*Little or Not at all*) to 5 (*More is not possible*). Adolescents addressed the items with respect to mother and father separately, and a mean score for each timepoint was computed, such that higher scores indicate higher parent-adolescent conflict. An example item is “How much do you and your mother/father get upset with or mad at each other?”. Cronbach's α ranged from .89 to .95 across timepoints.

Level of expressed emotion was assessed with the Dutch version of the Level of Expressed Emotion (LEE) scale (Gerlsma and Hale, 1997). This scale consists of 38 items, which assess lack of emotional support, intrusiveness, irritation, and criticism (Hale et al., 2016). The items are addressed on a 4-point Likert scale, from 1 (*Untrue*) to 4 (*True*), and a mean score was used such that high LEE scores correspond to high levels of perceived parental lack of emotional support, intrusiveness, irritation, and criticism. Adolescents responded to the items with respect to both of their parents. Example items are (“My parents:”) “Accuse me of exaggerating when I say I'm unwell”, and “Are critical of me”. Cronbach's α for the total scale ranged from .81 to .95 across timepoints.

Finally, peer victimization was assessed with the self-report of Aggression and Social Behavior Questionnaire (Morales and Crick, 1998), which assesses interpersonal aggression. Specifically, seven items assessing relational and physical aggression were used as a measure of victimization (example items: “Others try to make me do things by threatening me physically” and “Others say bad things about me behind my back”); addressed on a seven-point Likert-scale, ranging from 1 (*not at all true*) to 7 (*very true*). Prior research indicates good reliability and validity (Linder et al., 2002). In this sample, psychometric properties were acceptable, with Cronbach's α ranging between 0.73 and 0.87 across timepoints for physical victimization, and between 0.78 and 0.86 for relational victimization. Relational and physical subscales showed considerable overlap (*r* = 0.59) and were thus averaged into a total score representing adolescent victimization (Cronbach's α between 0.92 and 0.95).

#### 2.2.2 Depressive symptoms

Depressive symptoms were assessed using the Reynolds Adolescent Depression Scale - 2nd version (Reynolds, 2002). This scale consists of 23 adolescent-reported items, addressed on a scale from 1 (*Almost Never*) to 4 (*Usually*), including items for negative self-evaluation (e.g., “I feel I am bad”), dysphoric mood (e.g., “I feel sad”), and somatic complaints (e.g., “I feel tired”). A mean score of depressive symptoms was used, for which Cronbach's α ranged from .90 to .95 across timepoints. The RADS (Krefetz et al., 2002), and has been recommended as the tool of choice (Li et al., 2025).

#### 2.2.3 DNA methylation indices of CRP

DNA was extracted from saliva using the Oragene DNA kit (DNA Genotek). After bisulphite conversion using ZIMO kits, DNA methylation profiling was performed using the HumanMethylation 450K (age 17), and EPICv1 (age 25) BeadChip Arrays (Illumina, San Diego, CA). Preprocessing of methylation data was performed separately for age 17 and age 25 sample batches. We removed bad quality probes (detection *p*-value >0.01), as well as sex chromosome, cross-reactive, and SNP-containing probes and performed quantile normalization using the R package *minfi* (Hansen et al., 2022). We excluded one sample at age 17 and four samples at age 25 because their mean detection *p*-value was >0.01. In addition, we excluded seven samples at age 17 and two samples at age 25 because of a mismatch between reported and genetically inferred sex. In total, this resulted in 412 samples at age 17 and 231 samples at age 25 with DNAm data available post-QC.

For each individual, three DNAm predictors for CRP levels were calculated based on different EWAS studies of CRP conducted in blood: (1) Ligthart index: seven CpG's which related to serum CRP in an EWAS of in 8863 Europeans and which also related to whole-blood gene expression in *cis* (Barker et al., 2018; Ligthart et al., 2016), (2) Wielscher index: 1511 CpGs from the most recent EWAS of CRP in 22,774 individuals of different ancestries (Wielscher et al., 2022), and (3) Hillary index: 1469 CpGs from a novel elastic net regression-based predictor trained in 17,936 Europeans (Hillary et al., 2024). The Ligthart index has been previously applied in several mental health studies including in youth (Barker et al., 2018; Edmondson-Stait et al., 2022; Raffington et al., 2023; Suleri et al., 2024), whereas the Wielscher index has only been studied once in relation to depression in older adults (Verschoor et al., 2023). This is the first study applying the novel Hillary index to study mental health. In short, significant CpGs and their effect sizes were extracted from the original association study. Post-QC methylation β values for each CpG were multiplied by their effect size and summed to get DNAm indices. Only CpG sites for which data were available post-QC were included: 6 of 7 CpGs for the Ligthart index at both time points; 1443 of 1511 (age 17) and 1409 of 1511 (age 25) for the Wielscher index; and 1318 of 1468 (age 17) and 1420 of 1468 (age 25) for the Hillary index. Single CpG methylation values were standardized before calculating the Wielscher and Hillary index, but not for the Ligthart index, aligning with data preprocessing in the original studies. Final summed scores were standardized before performing integrated analyses between the two timepoints (i.e. the two batches measured on different array versions), to mitigate technical sources of systematic differences between timepoints. To evaluate the relation between the DNAm indices of CRP and cell type composition, we estimated the fraction of leukocytes versus epithelial cells from DNAm data using the saliva reference panel for studies in children by Middleton et al. (2022) using the *ewastools* R package (Just & Heiss, 2025).

#### 2.2.4 Covariates sensitivity analyses

**Gender**. Participants self-reported their gender in a binary format (0 – *male,* 1 – female).

**Socioeconomic Status**. Socio-economic status (SES) was based on parents' job level (Statistics Netherlands, 1993). If at least one of the parents' jobs was classified as medium (e.g., police officer, physician's assistant) or high level (e.g., doctor, scientist, high school teacher), SES in the sample was mostly medium/high (91%), whereas the rest of the sample was classified as low SES (9%, e.g., construction worker, janitor, truck driver).

**Smoking**. Participants indicated whether they smoked, and their responses were rescaled to a binary variable indicating never smoked (0 – *Never smoked*, 1 – *Smoked*).

### 2.3 Analytic plan

Detailed research questions, hypotheses and the analytic plan were pre-registered: https://osf.io/t2cp4/overview?view_only=8db91c0cb9f44a15915dc49c7e80d990. In short, as preliminary steps, Confirmatory Factor Analyses (CFAs) were conducted to substantiate the measurement of interpersonal stress as a latent variable comprised of repeated assessments of negative parenting (psychological control, conflict, level of expressed emotion) and victimization. The main analyses addressed three research questions: “Does interpersonal stress predict inflammation?” (RQ1), “Does inflammation predict depressive symptoms?” (RQ2) and “Does inflammation mediate the association between inflammation and depressive symptoms?”. Three Structural Equation Models were applied for each research question: two models which used either late adolescence or young adulthood assesment of DNAm indices of CRP, and a third model where the repeated assessments were, combined into a “mean” and “change” score using Latent Congruence Modeling (LCM, Cheung, 2009). Each of the nine models was applied to all three DNAm indices of CRP, resulting in 27 models in total. If significant effects emerged in any of these models, sensitivity analysis were run controlling for adolescent gender, smoking, and parental socioeconomic status. We applied False Discovery Rate (FDR)-based multiple testing correction to the p-values of target effects (e.g. DNAm score regressed on depressive symptoms) across the three DNAm indices of CRP. Two approaches were used to estimate model-based statistical power (based on number of variables and model complexity), which indicated satisfactory statistical power. For more details, please see https://osf.io/t2cp4/overview?view_only=8db91c0cb9f44a15915dc49c7e80d990.

#### 2.3.1 Deviations from the pre-registered analytic plan

In addition to the pre-registered analyses described above, we ran exploratory analyses whereby we separated interpersonal stress into family-based stress (negative parenting) and peer-based stress (peer victimization) and used these as two related predictors. In the results section below, these analyses are presented separately from the pre-registered analyses. During the review process, two further explorative analyses were performed. Firstly, the three DNAm indices op CRP were combined into one latent factor, aiming to improve reliability by evaluating the signal shared across indices. Secondly, as an alternative to averaging the scores reported for both parents, negative parenting was calculated using the higher of the two parents' scores on negative interactions and psychological control (i.e., the “harsher” parent), to reveal potential one-parent-based stress effects. The results of these analyses (available upon request) further support the conclusions of this study and are briefly presented below.

## 3 Results

### 3.1 Confirmatory Factor Analyses (CFAs)

Two CFAs examined the factor structure of interpersonal stress and depressive symptoms, repeatedly assessed from age 13 to 17 years. The fit was good for both models, all item standardized factor loadings were high (>.60), and composite reliability was .846 for interpersonal stress and .896 for depressive symptoms (Supplementary Tables S2–S3). For our exploratory analyses, we performed a CFA of interpersonal stress based on the highest scores out of both parents (“harsher” parent) only, as compared to averaging scores reported for mother and father. This showed worse properties (composite reliability .670, lower standardized factor loadings), compared to the original analyses. Additionally, we modelled a latent factor of the three DNAm indices of CRP (Ligthart, Hillary, Wielsher), which showed good internal consistency (composite reliability .87 for age 17 DNAm, and .90 for age 25 DNAm, all standardized loadings >.69).

### 3.2 Interpersonal stress, inflammation and depressive symptoms

Table S1 presents the means, standard deviations, and bivariate correlations among the study variables. Zero-order correlations between individual measurements indicated positive correlations between (i) interpersonal stress and depressive symptoms, (ii) the three DNAm indices of CRP with each other (0.58-0.83 within timepoint), and (iii) both interpersonal stress and depressive symptoms in adolescence with DNAm indices of CRP.

**Fig. 2:**
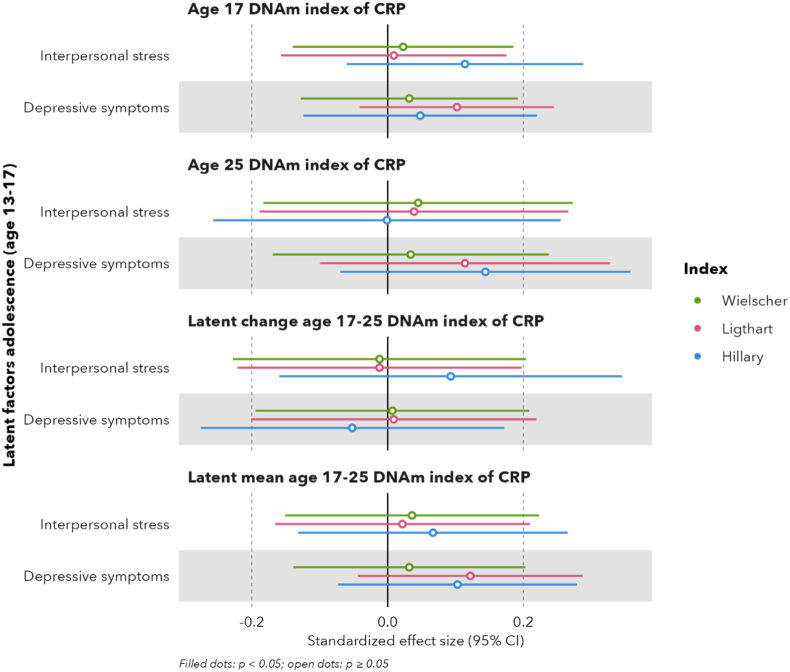
**Standardized parameter estimates for RQ1.** Structural equation models of Interpersonal Stress and Depressive Symptoms in adolescence (from age 13 to 17) predicting DNAm indices of CRP at age 17, age 25, or mean level and change from age 17 to 25. Filled dots indicate nominal p-values (uncorrected) < 0.05, open dots p > 0.05.

**Fig. 3:**
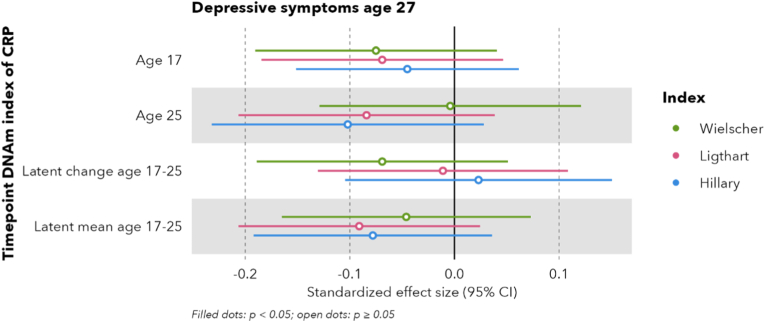
**Standardized parameter estimates for RQ2.** Structural equation models of DNAm indices of CRP at 17, 25, or mean level and change from age 17 to 25 predicting Depressive Symptoms at age 27. Filled dots indicate nominal p-values (uncorrected) < 0.05, open dots p > 0.05.

All 27 structural equation models showed good fit, based on at least two of the four fit indices. Across all models, no significant effects were found. Neither interpersonal stress nor depressive symptoms throughout adolescence predicted levels of any of the three DNAm indices of CRP in adolescence or young adulthood (RQ1, see Fig. 2 and Supplementary Table S5). DNAm indices of CRP did not predict changes in depressive symptoms from adolescence to young adulthood (RQ2, see Fig. 3, Supplementary Table S6) and did not mediate the effects of interpersonal stress throughout adolescence on depressive symptoms in young adulthood, or the carry-over effects of depressive symptoms from adolescence to young adulthood (RQ3, see Supplementary Table S7). Exploratory models using a latent factor of the three DNAm indices of CRP combined generally showed slightly improved fit and did not substantially alter any of these results, further supporting our null findings.

### 3.3 Family-based versus peer-based stress and inflammation

The CFAs for negative parenting and victimization as separate measures showed good measurement properties, indicated by improved fit indices (bottom half of Table S2), high standardized item loadings (all >.55), and good composite reliability estimates, (.786, and .800 for negative parenting and victimization, respectively). Table S4 presents the means, standard deviations, and bivariate correlations for the separate stress dimensions with other study variables. Notably, peer victimization was not associated with any of the DNAm indices of CRP, whereas all aspects of negative parenting (parent-adolescent conflict, parental psychological control, and level of expressed emotion-reverse coded) showed positive associations with the DNAm indices. Re-specifying negative parenting based on the highest parent score (“harsher” parent), as compared to averaging the scores for mother and father, resulted in slightly worse but still good fit, slightly weaker standardized factor loadings (all >.52), and lower composite reliability (.748).

**Fig. 4:**
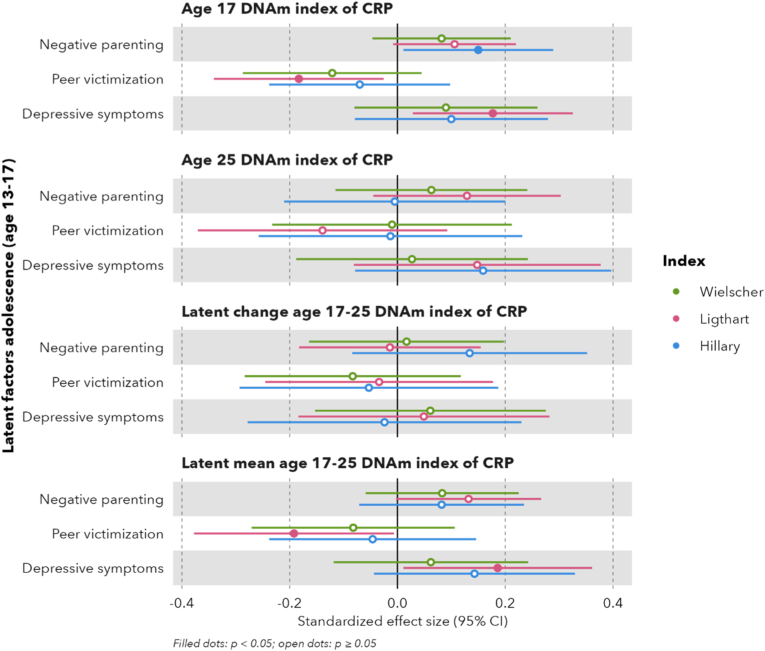
**Standardized parameter estimates for the exploratory models for RQ1.** Structural equation models for Negative Parenting and Peer Victimization separately, predicting DNAm CRP at age 17, age 25, or mean level and change from age 17 to 25. Filled dots indicate nominal p-values (uncorrected) < 0.05, open dots p > 0.05.

The exploratory analyses modelling negative parenting and peer victimization separately revealed a few significant effects (see Fig. 4 and Supplementary Tables S8–S9). Negative parenting had a significant positive effect on only the age 17 Hillary DNAm index of CRP, *β* = .15, *p* = .035; the same effect was positive and of similar magnitude but with a “trend-level” significance for the Ligthart index, *β* = .11, *p* = .067. Peer victimization had a negative effect, and depressive symptoms had a positive effect on the age 17 Ligthart DNAm index CRP (*β* = −.18, *p* = .023, and *β* = .18, *p* = .019), respectively. Finally, peer victimization and depressive symptoms had a significant negative (*β* = −.19, *p* = .042) and positive effect (*β* = .19, *p* = .037), respectively, on the average level of DNAm indices of CRP across the two measurements in the Latent Congruence Model part of RQ1, only for the Ligthart index (Fig. 4). When modeling negative parenting based on the highest parent score only (“harsher parent”), this gave similar but slightly stronger results. Namely, significant associations emerged for “harsher parent”-based negative parenting with age 17 DNAm indices of CRP for the Ligthart (*β* = .15, *p* = .015) and Hillary (*β* = .15, *p* = .037) index, and at age 25 only for the Ligthart index (*β* = .21, *p* = .012).

Given that some significant paths emerged, we ran sensitivity analyses controlling for sex, smoking and socioeconomic status. The trend-level positive effect of negative parenting on the Ligthart DNAm index of CRP at age 17 changed to *β* = .11, *p* = .050 and the effect on the Hillary DNAm index of CRP remained significant, *β* = .15, *p* = .020. The negative effect of peer victimization as well as the positive effect of depressive symptoms on the average level of DNAm of CRP for the Ligthart score turned non-significant, *β* = .0009, *p* = .916, and *β* = −.095, *p* = .280, respectively, while the effect of negative parenting turned positive, *β* = .130, *p* = .042. Finally, the effects of negative parenting based on the “harsher parent” only on DNAm indices of CRP remained significant upon adjusting for sex, smoking and socioeconomic status, but only the effect on age 17 Ligthart DNAm index of CRP survived multiple testing correction (FDR *p* < 0.05).

## 4 Discussion

In the current study, we investigated the longitudinal links between interpersonal stress during adolescence, epigenetic indices of inflammation in late adolescence and young adulthood, and depressive symptoms in young adulthood. Overall, no significant effects were found in the structural equation models, even though we observed weak zero-order positive correlations between either interpersonal stress or depressive symptoms with DNAm indices of CRP in late adolescence. However, when analysing family-based and peer-based interpersonal stress separately, few nominally significant associations emerged only for family-based stress with inflammation.

### 4.1 Interpersonal stress and inflammation

Contrary to our pre-registered hypotheses, we did not find evidence supporting that cumulative, interpersonal stress throughout adolescence predicts DNAm indices of inflammation during late adolescence or young adulthood. Two methodological decisions might account for this contrast with previous findings.

First, most prior studies measured protein levels of inflammatory markers such as CRP directly from blood, and not DNAm indices that proxy systemic inflammation from saliva. The few studies that did use DNAm to show links between interpersonal stress and inflammation used a “candidate-gene” approach, testing a limited number of CpGs (e.g., 8, or 222, in Beach et al., 2015; McDade et al., 2017, respectively) located in inflammatory genes. However, candidate-gene approaches have been criticized for substantially high Type I error rates (Shabalin et al., 2015), whereas composite epigenetic indices have been proposed to move the field forward (Cecil et al., 2023). In the current study, we applied such composite DNAm indices based on epigenome-wide association studies of CRP, which capture covariance across genomic loci. Future research may develop similar indices for additional inflammatory markers (e.g. TNF-α, IL-6) to improve understanding of specific inflammatory pathways.

Second, most studies to date on the link between interpersonal stress and inflammation focused on narrowly defined conceptualizations of interpersonal stress, such as bullying (Copeland et al., 2014), the absence of a parent (McDade et al., 2017), or protective parenting (Beach et al., 2015, p. 201). In this study, we conceptualized interpersonal stress broadly, as a composite variable comprised of several stressful experiences within and outside the family, accumulated across years. While this methodological choice aimed to reduce the number of tests and capture potentially cumulative effects, it may have obscured underlying distinctive effects. For example, in a study testing several childhood adversity dimensions, different interpersonal stress indicators were found to have varying links with levels of DNAm (Copeland et al., 2022). Therefore, in additional exploratory analyses, we decided to examine family- and peer-based stress separately.

Although both the Ligthart and Wielscher DNAm indices of CRP were adjusted for immune cell type composition upon training in blood, we observed that all three DNAm indices of CRP were correlated with the proportion of immune cells in saliva (see Supplementary Fig. S11). Thus, shifts in immune cell proportions arising from cell proliferation and migration during immune activation likely contribute to the immune biomarker potential of DNAm as captured in DNAm indices of CRP, also in saliva.

### 4.2 Family-based vs peer-based stress and inflammation

When investigating both family-based and peer-based stress separately, peer-based stress showed essentially no bivariate correlations with any of the DNAm indices of CRP. In contrast, the three measures of negative parenting correlated positively with two DNAm indices. Accordingly, adolescents who experienced more negative parenting from ages 13 to age 17 tended to show a higher DNAm index of inflammation at age 17 (for one out of three indices).

Our results extend past literature by showing that the association between family-based stress and inflammation is reflected in DNAm indices of CRP from saliva. Also, our findings build on a study in the same cohort, which failed to find evidence that negative parenting is linked to epigenetic age acceleration (Mastrotheodoros et al., 2023). Our new findings suggest that negative parenting might still be biologically embedded in the methylome, affecting immune-related rather than aging processes. Notably, the association between negative parenting and DNAm indices of inflammation appeared to depend on the timing of inflammation measurement relative to the experienced stress, as it appeared in late adolescence but not in young adulthood—although a trend in the same direction was observed in young adulthood. Unobserved pro-inflammatory exposures in young adulthood may have masked any long-term effect of negative parenting. The within-person stability of DNAm indices of CRP between the two timepoints was only moderate (ICC .30-.57, see Supplementary Table S10). Future studies should further explore measurement timing-dependent effects of stress on inflammation, paying attention to time-varying environmental confounders in a larger study sample.

In contrast to our hypothesis, the latent factor of peer victimization was negatively associated with one out of three DNAm indices of CRP. However, this negative effect did not remain significant upon adjusting for potential confounders, while the association between negative parenting and DNAm indices of CRP remained significant. It is notable that victimization was overall very low (on average across timepoints 68% of the participants responded “not at all” to the victimization items), and its distribution was skewed in this sample. Furthermore, past studies on the link between peer victimization and inflammation using blood-based measures of CRP directly assessed bullying, whereas we conceptualized peer victimization as experiencing interpersonal aggression.

Taken together, the exploratory analyses tentatively suggest differences in the effect of peer victimization versus negative parenting on DNAm indices of inflammation. These differences can be explained by biosocial models of development (e.g., Bronfenbrenner and Morris, 2006). As family is a more proximal context compared to peer relationships, issues in this domain might be more strongly linked with inflammation. For example, negative parenting may reflect stress exposures not only in adolescence but also in early childhood. Furthermore, a suboptimal family environment might be related to youth's health behaviours (Philips et al., 2014), such as nutrition, exercise, and sleep, impacting inflammation. In contrast, victimization by peers takes place in a more distal, specific social context.

### 4.3 Depressive symptoms and inflammation

We found no evidence supporting a positive relation between inflammation during adolescence and depressive symptoms in young adulthood. Previous studies using epigenetic indices of CRP, like this study, are inconclusive, reviewed elsewhere (Schalkwijk et al., 2026). Methodological choices might account for differences between studies, such as age of the study population, assessment of depressive symptoms (or clinical depression), and cross-sectional versus longitudinal study designs.

### 4.4 Strengths and limitations

Strengths of this study are the repeated measurements of DNAm, providing insights into intrapersonal dynamics and age-dependency of relationships, the preregistered methodological approach, and the use of three different DNAm markers of CRP, including two novel ones (Wielscher, Hillary), not studied in relation to stress or mental health before. A potential limitation is the power of our sample to detect small effects, even though the model-based power was satisfactory. Future studies with repeated measures and larger samples could provide further insights into the directionality and strength of associations between interpersonal stress, DNAm indices of inflammation, and depression. Further, our study design did not allow for investigating changes in DNAm in relation to stress or depressive symptoms throughout adolescence, since we only had one measurement of DNAm in adolescence. Future research with repeated assessments of all three concepts throughout adolescence could clarify these early dynamics further. Furthermore, although interpersonal stress in adolescence is a well-established risk factor for depressive symptoms, no such association was observed in this study, which limited our ability to explore inflammation-related mediation effects.

Another potential limitation of this study is the application of adult-based blood-trained DNAm indices to saliva from youth, since tissue and age translation of DNAm is a well-known challenge. That said, the blood-based Ligthart DNAm index of CRP was previously applied using saliva DNAm, validated to modestly correlate with serum levels of CRP and negative neighborhood dynamics in youth (Mrug et al., 2022) and to relate to perinatal inflammation in neonates (Conole et al., 2023), which supports its use in both saliva samples and in children. Unfortunately, no serum CRP levels were available for validation of the DNAm indices in our study. The field of DNAm indices of inflammation is new and emerging; therefore, having both DNAm indices of CRP and serum levels of CRP would help illuminate the comparative value of both measures in the context of our study.

A technical limitation comes from the fact that the two timepoints of DNAm sampling were processed separately and on different methylation array versions. Future studies with repeated samples from one individual analyzed on the same methylation plate and array version may to help further distinguish age differences in DNAm indices of CRP from technical variation. A further limitation is that no data were available on overweight (BMI) or inflammatory conditions (e.g. infection, autoimmune disease) that may impact measures of inflammation.

## 5 Conclusion

To conclude, our main analyses do not support an association between interpersonal stress or epigenetic indices of inflammation with later depressive symptoms. Our exploratory findings do suggest that negative parenting during adolescence may contribute to chronic inflammation, which was not found for peer victimization. The present study contributes to characterization of novel DNAm indices of CRP and highlights their potential for developmental mental health research. Further research is needed to disentangle the complex multidirectional relations between stress, epigenetics, inflammation, and depression, including potential immune-mediated timing effects across development.

## Ethics statement

Informed consent was obtained from all individual participants included in the study, for each timepoint separately after explaining their role and their rights in the study and before starting data collection. The Medical Ethical Committee of the Utrecht Medical Centre, The Netherlands, approved this study on the 29th of September 2005 (approval number: 05/159-K).

## CRediT authorship contribution statement

**Stefanos Mastrotheodoros:** Conceptualization, Data curation, Formal analysis, Investigation, Methodology, Project administration, Visualization, Writing – original draft, Writing – review & editing. **Winni Schalkwijk:** Conceptualization, Formal analysis, Investigation, Methodology, Project administration, Visualization, Writing – original draft, Writing – review & editing. **Michelle de Groot:** Data curation. **Sinan Gülöksüz:** Funding acquisition, Writing – review & editing. **Stefanie A. Nelemans:** Conceptualization, Methodology, Writing – review & editing. **Jim van Os:** Funding acquisition. **Bart P.F. Rutten:** Funding acquisition, Writing – review & editing. **Wim Meeus:** Funding acquisition, Resources, Writing – review & editing. **Marco P. Boks:** Conceptualization, Funding acquisition, Methodology, Project administration, Supervision, Writing – review & editing. **Susan Branje:** Conceptualization, Funding acquisition, Methodology, Writing – review & editing.

## Declaration of competing interest

The authors declare that they have no conflict of interest.

## Data Availability

The RADAR dataset is available in the DANS repository, https://doi.org/10.17026/dans-zrb-v5wp. Due to ethical and GDPR restrictions, the data are not publicly available. However, the dataset can be obtained from the authors upon reasonable request and with permission from the RADAR management team.
