# Supplementary material for: Interpersonal stress, epigenetic indices of inflammation, and depressive symptoms: Longitudinal associations from adolescence to young adulthood
Source: Neurobiol Stress. 2026 Jun 22;43:100832. doi: 10.1016/j.ynstr.2026.100832 (PMC13320348; doi:10.1016/j.ynstr.2026.100832)
Supplement: Multimedia component 1 [file mmc1.docx]

**Appendix with Supplementary Tables**

- **Table S1**. Means, standard deviations, and correlations of the main study variables.
- **Table S2.** Fit indices of all models in the study.
- **Table S3.** Standardized factor loadings of the four indicators of interpersonal stress across five waves.
- **Table S4**. Exploratory analyses: means, standard deviations, and bivariate correlations of study variables.
- **Table S5.** Standardized parameter estimates for RQ1. Structural equation models of Interpersonal Stress or Depressive Symptoms in adolescence (from age 13 to 17) predicting DNAm indices of CRP at age 17, age 25, or mean level and change from age 17 to 25.
- **Table S6**. Standardized parameter estimates for RQ2. Structural equation models of DNAm indices of CRP at 17, 25, or mean level and change from age 17 to 25 predicting Depressive Symptoms at age 27.
- **Table S7.** Standardized parameter estimates for RQ3. Structural equation mediation models of Interpersonal Stress and Depressive Symptoms from age 13 to 17 predicting age 27 Depressive Symptoms, mediated by DNAm CRP at 17, 25, or mean level or change from 17 to 25.
- **Table S8.** Standardized parameter estimates for the exploratory models for RQ1. Structural equation models for Negative Parenting and Peer Victimization separately, predicting DNAm CRP at age 17, age 25, or mean level and change from age 17 to 25.
- **Table S9**. Standardized parameter estimates for the exploratory models for RQ3. Structural equation models of Negative Parenting and Peer Victimization separately, as well as Depressive Symptoms from age 13 to 17, predicting age 27 Depressive Symptoms, mediated by DNAm CRP at age 17, age 25, or mean level and change from 17 to 25.
- **Table S10**. Intraclass correlation (ICC) of repeated measurements of DNAm indices of CRP.
- **Figure S11**. Covariance plots for the DNAm-estimated proportion of leukocytes in saliva with DNAm indices of CRP, age, interpersonal stress, and depression.

**Table *S*1. Means, standard deviations, and correlations of the main study variables*.*** *M*: mean; *SD*: standard deviation. Intstr: Interpersonal stress, a composite score comprised of negative parenting (psychological control, parent-adolescent conflict, parental criticism) and peer victimization. Depr: Depressive symptoms. Numbers next to variable names indicate age at assessment. Age 1717: first measurement of DNAm (some months after study visit). Age 2525: second measurement of DNAm (~ 7 years after W5). DNA methylation indices of CRP have been scaled within each timepoint. * *p* < .05. ** *p* < .01.

| Variable | *M* | *SD* | 1 | 2 | 3 | 4 | 5 | 6 | 7 | 8 | 9 | 10 | 11 | 12 | 13 | 14 | 15 | 16 |
| --- | --- | --- | --- | --- | --- | --- | --- | --- | --- | --- | --- | --- | --- | --- | --- | --- | --- | --- |
| 1. intstr13 | 0.00 | 0.50 |  |  |  |  |  |  |  |  |  |  |  |  |  |  |  |  |
| 2. intstr14 | -0.00 | 0.45 | .55** |  |  |  |  |  |  |  |  |  |  |  |  |  |  |  |
| 3. intstr15 | -0.00 | 0.46 | .46** | .59** |  |  |  |  |  |  |  |  |  |  |  |  |  |  |
| 4. intstr16 | -0.00 | 0.44 | .43** | .52** | .66** |  |  |  |  |  |  |  |  |  |  |  |  |  |
| 5. intstr17 | -0.00 | 0.45 | .35** | .45** | .55** | .66** |  |  |  |  |  |  |  |  |  |  |  |  |
| 6. depr13 | 1.64 | 0.40 | .47** | .36** | .34** | .32** | .29** |  |  |  |  |  |  |  |  |  |  |  |
| 7. depr14 | 1.50 | 0.50 | .33** | .49** | .43** | .37** | .36** | .54** |  |  |  |  |  |  |  |  |  |  |
| 8. depr15 | 1.53 | 0.52 | .29** | .38** | .52** | .42** | .34** | .53** | .67** |  |  |  |  |  |  |  |  |  |
| 9. depr16 | 1.56 | 0.54 | .34** | .36** | .43** | .47** | .40** | .51** | .64** | .72** |  |  |  |  |  |  |  |  |
| 10. depr17 | 1.54 | 0.51 | .30** | .35** | .41** | .42** | .46** | .47** | .55** | .65** | .76** |  |  |  |  |  |  |  |
| 11. depr25 | 1.71 | 0.46 | .22** | .23** | .29** | .28** | .25** | .36** | .37** | .41** | .44** | .45** |  |  |  |  |  |  |
| 12. ligthart17 | -0.01 | 1.01 | -.04 | .09 | .11* | .05 | .12* | .04 | .08 | .14** | .10 | .11* | -.01 |  |  |  |  |  |
| 13. ligthart25 | -0.00 | 1.00 | -.09 | .10 | .13 | .08 | .16* | .00 | .10 | .11 | .15* | .15* | -.01 | .42** |  |  |  |  |
| 14. wielscher17 | -0.01 | 0.99 | -.02 | .09 | .07 | .02 | .05 | .01 | .05 | .08 | .06 | .02 | -.06 | .81** | .31** |  |  |  |
| 15.wielscher25 | 0.00 | 1.00 | -.04 | .07 | .07 | .03 | .12 | .01 | .04 | .03 | .08 | .08 | .02 | .30** | .83** | .35** |  |  |
| 16. hillary17 | -0.02 | 0.99 | .07 | .12* | .12* | .11* | .11* | .04 | .08 | .14** | .12* | .09 | .01 | .58** | .32** | .66** | .25** |  |
| 17. hillary25 | -0.00 | 1.00 | -.06 | .08 | .09 | .05 | .11 | .07 | .15* | .12 | .10 | .12 | -.03 | .34** | .70** | .31** | .70** | .38** |

**Table S2. Fit indices of all models in the study.** DNAm 17: DNA methylation score at age 17 years; DNAm 25: DNA methylation score at age 25 years; LCS: Latent Congruence Score. Numbers next to variable names indicate age at assessment.

|  | *χ^2^* | *df* | CFI | TLI | RMSEA[90%CI] | SRMR |
| --- | --- | --- | --- | --- | --- | --- |
| CFA |  |  |  |  |  |  |
| Interpersonal stress | 44.921 | 5 | .952 | .903 | .139 [.103-.177] | .037 |
| Depressive symptoms | 46.202 | 5 | .965 | .929 | .140 [.104-.179] | .031 |
| Research Question 1: Interpersonal Stress & Depressive Symptoms 🡪 DNAm |  |  |  |  |  |  |
| 1a.1 Ligthart 17 | 202.537 | 40 | .922 | .892 | .104 [.089-.119] | .044 |
| 1a.2 Hillary 17 | 196.516 | 40 | .925 | .897 | .101 [.086-.117] | .043 |
| 1a.3 Wielscher 17 | 181.471 | 40 | .931 | .905 | .098 [.083-.113] | .041 |
| 1b.1 Ligthart 25 | 187.716 | 40 | .924 | .895 | .103 [.087-.120] | .049 |
| 1b.2 Hillary 25 | 177.199 | 40 | .934 | .909 | .096 [.078-.113] | .043 |
| 1b.3 Wielscher 25 | 182.417 | 40 | .932 | .906 | .097 [.081-.114] | .043 |
| 1c.1 Ligthart LCS | 202.643 | 48 | .925 | .897 | .095 [.080-.110] | .047 |
| 1c.2 Hillary LCS | 184.793 | 48 | .938 | .914 | .086 [.070-.102] | .040 |
| 1c.3 Wielscher LCS | 194.666 | 48 | .932 | .907 | .089 [.074-.105] | .042 |
| Research Question 2: DNAm 🡪 Depressive Symptoms 171 |  |  |  |  |  |  |
| 2a.1 Ligthart 17 | 42.631 | 14 | .971 | .957 | .073 [.049-.099] | .042 |
| 2a.2 Hillary 17 | 42.125 | 14 | .971 | .956 | .073 [.049-.099] | .045 |
| 2a.3 Wielscher 17 | 39.731 | 14 | .974 | .960 | .070 [.045-.096] | .030 |
| 2b.1 Ligthart 25 | 42.324 | 14 | .970 | .955 | .074 [.049-.101] | .050 |
| 2b.2 Hillary 25 | 39.898 | 14 | .972 | .959 | .071 [.046-.098] | .050 |
| 2b.3 Wielscher 25 | 38.924 | 14 | .974 | .961 | .069 [.044-.095] | .033 |
| 2c.1 Ligthart LCS | 46.643 | 17 | .972 | .954 | .067 [.044-.090] | .031 |
| 2c.2 Hillary LCS | 40.544 | 17 | .977 | .961 | .060 [.037-.084] | .025 |
| 2c.3 Wielscher LCS | 43.572 | 17 | .974 | .958 | .063 [.040-.086] | .027 |
| Research Question 3: Interpersonal Stress & Depressive Symptoms 🡪 DNAm 🡪 Depressive Symptoms 171 |  |  |  |  |  |  |
| 3a.1 Ligthart 17 | 226.074 | 48 | .932 | .906 | .091 [.078-.104] | .039 |
| 3a.2 Hillary 17 | 219.287 | 48 | .935 | .911 | .089 [.076-.102] | .038 |
| 3a.3 Wielscher 17 | 223.950 | 48 | .933 | .908 | .090 [.077-.104] | .039 |
| 3b.1 Ligthart 25 | 231.154 | 48 | .927 | .899 | .095 [.081-.109] | .045 |
| 3b.2 Hillary 25 | 222.170 | 48 | .935 | .911 | .089 [.074-.103] | .040 |
| 3b.3 Wielscher 25 | 222.974 | 48 | .934 | .909 | .090 [.075-.104] | .041 |
| 3c.1 Ligthart LCS | 242.228 | 56 | .928 | .899 | .088 [.075-.102] | .044 |
| 3c.2 Hillary LCS | 226.484 | 56 | .938 | .914 | .081 [.067-.095] | .038 |
| 3c.3 Wielscher LCS | 231.735 | 56 | .934 | .909 | .083 [.070-.097] | .039 |
| Exploratory Analyses* | | | | | | |
| CFA |  |  |  |  |  |  |
| Negative parenting | 12.949 | 4 | .987 | .968 | .071 [.028-.117] | .024 |
| Peer victimization | 7.169 | 3 | .995 | .982 | .061 [.000-.119] | .016 |
| Research Question 1: Negative Parenting, Peer Victimization, & Depressive Symptoms 🡪 DNAm |  |  |  |  |  |  |
| 1a.1 Ligthart 17 | 296.649 | 95 | .928 | .909 | .075 [.065-.086] | .044 |
| 1a.2 Hillary 17 | 289.344 | 95 | .931 | .913 | .073 [.063-.084] | .042 |
| 1a.3 Wielscher 17 | 298.852 | 95 | .927 | .907 | .076 [.065-.086] | .043 |
| 1b.1 Ligthart 25 | 301.638 | 95 | .923 | .903 | .078 [.067-.089] | .048 |
| 1b.2 Hillary 25 | 288.960 | 95 | .932 | .914 | .073 [.061-.084] | .044 |
| 1b.3 Wielscher 25 | 294.578 | 95 | .930 | .911 | .074 [.063-.085] | .044 |
| 1c.1 Ligthart LCS | 320.606 | 107 | .925 | .904 | .073 [.063-.084] | .047 |
| 1c.2 Hillary LCS | 301.249 | 107 | .935 | .918 | .067 [.057-.078] | .042 |
| 1c.3 Wielscher LCS | 316.735 | 107 | .928 | .908 | .071 [.061-.082] | .044 |
| Research Question 3: Negative Parenting, Peer Victimization, & Depressive Symptoms 🡪 DNAm 🡪 Depressive Symptoms 17 |  |  |  |  |  |  |
| 3a.1 Ligthart 17 | 370.730 | 107 | .922 | .901 | .075 [.066-.084] | .043 |
| 3a.2 Hillary 17 | 363.130 | 107 | .925 | .905 | .074 [.065-.083] | .042 |
| 3a.3 Wielscher 17 | 372.625 | 107 | .921 | .900 | .076 [.067-.085] | .043 |
| 3b.1 Ligthart 25 | 376.158 | 107 | .918 | .896 | .077 [.068-.087] | .046 |
| 3b.2 Hillary 25 | 364.049 | 107 | .926 | .906 | .073 [.063-.083] | .043 |
| 3b.3 Wielscher 25 | 366.613 | 107 | .924 | .904 | .074 [.064-.084] | .043 |
| 3c.1 Ligthart LCS | 390.801 | 119 | .920 | .897 | .073 [.064-.082] | .045 |
| 3c.2 Hillary LCS | 372.182 | 119 | .929 | .909 | .068 [.059-.078] | .041 |
| 3c.3 Wielscher LCS | 384.850 | 119 | .923 | .901 | .071 [.062-.081] | .043 |

*Note:* ********Exploratory Analyses refer to non-preregistered analyses that used Negative Parenting and Peer Victimization as separate predictors, instead of the composite Interpersonal Stress; Research Question 2 analyses were not repeated in the Exploratory Analyses as they did not include interpersonal stress.*

**Table S3. Standardized factor loadings of the four indicators of interpersonal stress across five waves.**

| Interpersonal Stress Indicator | Wave 1 | Wave 2 | Wave 3 | Wave 4 | Wave 5 |
| --- | --- | --- | --- | --- | --- |
| Conflict | .662 | .734 | .700 | .692 | .743 |
| Psychological Control | .713 | .749 | .824 | .805 | .778 |
| Level of Expressed Emotion | -.706 | -.801 | -.822 | -.849 | -.823 |
| Peer Victimization | .413 | .459 | .482 | .419 | .381 |

*Note*. All factor loadings were significant, *p* < .001

**Table S4. Exploratory analyses: means, standard deviations, and bivariate correlations of study variables.**

| Variable | *M* | *SD* | 1 | 2 | 3 | 4 | 5 | 6 | 7 | 8 | 9 | 10 | 11 | 12 | 13 | 14 | 15 | 16 | 17 | 18 | 19 | 20 | 21 | 22 | 23 | 24 | 25 | 26 | 27 | 28 | 29 | 30 | 31 |
| --- | --- | --- | --- | --- | --- | --- | --- | --- | --- | --- | --- | --- | --- | --- | --- | --- | --- | --- | --- | --- | --- | --- | --- | --- | --- | --- | --- | --- | --- | --- | --- | --- | --- |
| 1. nr13 | -0.00 | 1.00 |  |  |  |  |  |  |  |  |  |  |  |  |  |  |  |  |  |  |  |  |  |  |  |  |  |  |  |  |  |  |  |
| 2. n14 | 0.00 | 1.00 | .65** |  |  |  |  |  |  |  |  |  |  |  |  |  |  |  |  |  |  |  |  |  |  |  |  |  |  |  |  |  |  |
| 3. nr15 | 0.00 | 1.00 | .52** | .63** |  |  |  |  |  |  |  |  |  |  |  |  |  |  |  |  |  |  |  |  |  |  |  |  |  |  |  |  |  |
| 4. nr16 | 0.00 | 1.00 | .46** | .59** | .64** |  |  |  |  |  |  |  |  |  |  |  |  |  |  |  |  |  |  |  |  |  |  |  |  |  |  |  |  |
| 5. nr17 | 0.00 | 1.00 | .46** | .54** | .62** | .71** |  |  |  |  |  |  |  |  |  |  |  |  |  |  |  |  |  |  |  |  |  |  |  |  |  |  |  |
| 6. pc13 | -0.00 | 1.00 | .46** | .34** | .29** | .28** | .26** |  |  |  |  |  |  |  |  |  |  |  |  |  |  |  |  |  |  |  |  |  |  |  |  |  |  |
| 7. pc14 | -0.00 | 1.00 | .44** | .56** | .43** | .39** | .37** | .44** |  |  |  |  |  |  |  |  |  |  |  |  |  |  |  |  |  |  |  |  |  |  |  |  |  |
| 8. pc15 | -0.00 | 1.00 | .33** | .38** | .57** | .45** | .40** | .48** | .57** |  |  |  |  |  |  |  |  |  |  |  |  |  |  |  |  |  |  |  |  |  |  |  |  |
| 9. pc16 | -0.00 | 1.00 | .30** | .35** | .46** | .56** | .45** | .48** | .53** | .70** |  |  |  |  |  |  |  |  |  |  |  |  |  |  |  |  |  |  |  |  |  |  |  |
| 10. pc17 | 0.00 | 1.00 | .32** | .32** | .44** | .46** | .58** | .39** | .44** | .61** | .69** |  |  |  |  |  |  |  |  |  |  |  |  |  |  |  |  |  |  |  |  |  |  |
| 11. le13 | -0.00 | 1.00 | -.47** | -.40** | -.36** | -.30** | -.30** | -.50** | -.38** | -.37** | -.36** | -.31** |  |  |  |  |  |  |  |  |  |  |  |  |  |  |  |  |  |  |  |  |  |
| 12. le14 | -0.00 | 1.00 | -.42** | -.58** | -.48** | -.42** | -.40** | -.44** | -.60** | -.55** | -.51** | -.41** | .63** |  |  |  |  |  |  |  |  |  |  |  |  |  |  |  |  |  |  |  |  |
| 13. le15 | 0.00 | 1.00 | -.29** | -.40** | -.59** | -.45** | -.42** | -.39** | -.44** | -.68** | -.61** | -.51** | .49** | .69** |  |  |  |  |  |  |  |  |  |  |  |  |  |  |  |  |  |  |  |
| 14. le16 | 0.00 | 1.00 | -.30** | -.39** | -.46** | -.59** | -.50** | -.37** | -.43** | -.55** | -.68** | -.53** | .45** | .66** | .72** |  |  |  |  |  |  |  |  |  |  |  |  |  |  |  |  |  |  |
| 15. le17 | -0.00 | 1.00 | -.34** | -.37** | -.44** | -.51** | -.62** | -.31** | -.35** | -.49** | -.58** | -.64** | .42** | .60** | .66** | .80** |  |  |  |  |  |  |  |  |  |  |  |  |  |  |  |  |  |
| 16. vi13 | -0.00 | 1.00 | .28** | .22** | .17** | .15** | .14** | .31** | .26** | .23** | .21** | .20** | -.28** | -.28** | -.20** | -.17** | -.21** |  |  |  |  |  |  |  |  |  |  |  |  |  |  |  |  |
| 17. vi14 | 0.00 | 1.00 | .30** | .33** | .27** | .20** | .21** | .30** | .32** | .34** | .26** | .26** | -.31** | -.38** | -.31** | -.29** | -.32** | .55** |  |  |  |  |  |  |  |  |  |  |  |  |  |  |  |
| 18. vi15 | 0.00 | 1.00 | .19** | .28** | .33** | .24** | .26** | .23** | .29** | .42** | .34** | .31** | -.26** | -.33** | -.37** | -.30** | -.30** | .50** | .58** |  |  |  |  |  |  |  |  |  |  |  |  |  |  |
| 19. vi16 | -0.00 | 1.00 | .21** | .22** | .29** | .26** | .26** | .22** | .27** | .34** | .34** | .33** | -.26** | -.31** | -.34** | -.37** | -.36** | .41** | .52** | .66** |  |  |  |  |  |  |  |  |  |  |  |  |  |
| 20. vi17 | -0.00 | 1.00 | .14** | .20** | .26** | .19** | .26** | .16** | .20** | .22** | .26** | .32** | -.24** | -.27** | -.29** | -.25** | -.32** | .40** | .44** | .52** | .61** |  |  |  |  |  |  |  |  |  |  |  |  |
| 21. de13 | 1.64 | 0.40 | .39** | .30** | .31** | .24** | .20** | .37** | .33** | .33** | .31** | .32** | -.36** | -.37** | -.36** | -.31** | -.32** | .51** | .39** | .34** | .31** | .32** |  |  |  |  |  |  |  |  |  |  |  |
| 22. de14 | 1.50 | 0.50 | .29** | .40** | .39** | .27** | .30** | .23** | .39** | .40** | .30** | .30** | -.22** | -.42** | -.40** | -.31** | -.28** | .32** | .53** | .41** | .37** | .31** | .54** |  |  |  |  |  |  |  |  |  |  |
| 23. de15 | 1.53 | 0.52 | .24** | .31** | .43** | .35** | .28** | .23** | .30** | .49** | .38** | .33** | -.23** | -.33** | -.45** | -.37** | -.28** | .29** | .40** | .48** | .38** | .28** | .53** | .67** |  |  |  |  |  |  |  |  |  |
| 24. de16 | 1.56 | 0.54 | .30** | .31** | .38** | .40** | .35** | .30** | .34** | .44** | .46** | .41** | -.23** | -.35** | -.42** | -.49** | -.40** | .26** | .35** | .40** | .45** | .35** | .51** | .64** | .72** |  |  |  |  |  |  |  |  |
| 25. de17 | 1.54 | 0.51 | .32** | .31** | .37** | .37** | .40** | .27** | .27** | .39** | .40** | .45** | -.24** | -.32** | -.37** | -.43** | -.44** | .22** | .37** | .37** | .41** | .40** | .47** | .55** | .65** | .76** |  |  |  |  |  |  |  |
| 26. de27 | 1.71 | 0.46 | .14** | .20** | .24** | .27** | .20** | .13* | .17** | .27** | .28** | .22** | -.06 | -.23** | -.21** | -.27** | -.24** | .21** | .28** | .24** | .20** | .23** | .36** | .37** | .41** | .44** | .45** |  |  |  |  |  |  |
| 27. Ligthart 17 | -0.01 | 0.00 | .12* | .13** | .12* | .07 | .09 | -.02 | .13* | .10 | .06 | .06 | -.14** | -.08 | -.05 | -.05 | .02 | -.04 | -.04 | .04 | .01 | .04 | .04 | .08 | .14** | .10 | .11* | -.01 |  |  |  |  |  |
| 28. Ligthart 25 | -0.01 | 0.00 | .05 | .07 | .04 | -.02 | .07 | .01 | .05 | .06 | -.01 | .06 | -.10 | .05 | .08 | .11 | .14* | -.13* | -.01 | .05 | .06 | .02 | .00 | .10 | .11 | .15* | .15* | -.01 | .42** |  |  |  |  |
| 29. Wielscher 17 | -11.28 | 757.24 | .07 | .11* | .06 | .07 | .05 | -.04 | .11* | .06 | .01 | -.01 | -.09 | -.04 | -.01 | -.05 | .06 | .00 | -.03 | .01 | .01 | -.00 | .01 | .05 | .08 | .06 | .02 | -.06 | .81** | .31** |  |  |  |
| 30. Wielscher 25 | 0.00 | 721.86 | .06 | .03 | -.02 | -.03 | .06 | -.00 | -.02 | -.01 | -.08 | -.02 | -.06 | .09 | .12 | .11 | .14* | -.06 | .02 | .05 | .05 | .03 | .01 | .04 | .03 | .08 | .08 | .02 | .30** | .83** | .35** |  |  |
| 31. Hillary 17 | -0.01 | 0.33 | .08 | .09 | .06 | .07 | .06 | -.00 | .13* | .12* | .12* | .08 | .02 | -.00 | -.03 | -.04 | .04 | .04 | -.01 | .07 | .05 | .02 | .04 | .08 | .14** | .12* | .09 | .01 | .58** | .32** | .66** | .25** |  |
| 32. Hillary 25 | 0.00 | 0.38 | .03 | .05 | -.03 | -.05 | .00 | .04 | .05 | .04 | .00 | .03 | -.12 | -.01 | .06 | .06 | .14* | -.05 | .05 | .10 | .06 | .04 | .07 | .15* | .12 | .10 | .12 | -.03 | .34** | .70** | .31** | .70** | .38** |

*Note.* *M*: mean; *SD*: standard deviation. nr: parent-adolescent conflict; pc: parental psychological control; le: parental level of expressed emotion; vi: peer victimization, de: Depressive symptoms. Numbers next to variable names indicate age at assessment. Age 17: first measurement of DNAm (some months after study visit). Age 25: second measurement of DNAm (~ 7 years after frist measurement of DNAm). DNA methylation indices of CRP have been scaled within each timepoint.

. * *p* < .05. ** *p* < .01.

Supplements continue on next page.

**Table S5. Standardized parameter estimates for RQ1.** Structural equation models of Interpersonal Stress or Depressive Symptoms in adolescence (from age 13 to 17) predicting DNAm indices of CRP at age 17, age 25, or mean level and change from age 17 to 25.

|  | Ligthart | | | Hillary | | | Wielscher | | |
| --- | --- | --- | --- | --- | --- | --- | --- | --- | --- |
|  | *β* | 95% CI | *p* | *β* | 95% CI | *p* | *β* | 95% CI | *p* |
|  | DNAm CRP age 17 | | | | | | | | |
| Interpersonal Stress age 13-17^1^ | .009 | -.157-.175 | .913 | .114 | -.060-.289 | .198 | .023 | -.139 -.186 | .780 |
| Depressive Symptoms age 13-17^2^ | .102 | -.041-.245 | .161 | .048 | -.124-.220 | .584 | .032 | -.128-.192 | .693 |
|  | DNAm CRP age 25 | | | | | | | | |
| Interpersonal Stress age 13-17^1^ | .039 | -.188-.267 | .737 | -.001 | -.257-.255 | .992 | .045 | -.183-.273 | .697 |
| Depressive Symptoms age 13-17^2^ | .114 | -.100-.327 | .296 | .144 | -.070-.358 | .187 | .034 | -.169-.238 | .741 |
|  | Latent Congruence Model | | | | | | | | |
|  | Congruence DNAm CRP age 17-25 | | | | | | | | |
| Interpersonal Stress Aage13-17^1^ | -.012 | -.222-.197 | .909 | .093 | -.160-.345 | .472 | -.012 | -.228-.204 | .913 |
| Depressive Symptoms age 13-17^2^ | .009 | -.201-.219 | .934 | -.052 | -.275-.172 | .651 | .007 | -.195-.208 | .948 |
|  | Mean level DNAm CRP age 17-25 | | | | | | | | |
| Interpersonal Stress age 13-17^1^ | .022 | -.165-.210 | .815 | .067 | -.131-.266 | .506 | .036 | -.151-.223 | .706 |
| Depressive Symptoms age 13-17^2^ | .122 | -.044-.288 | .150 | .103 | -.073-.279 | .252 | .032 | -.139-.203 | .712 |

*Note. The results of several models are summarized here. Each DNAm CRP score at both age 17 and age 25, as well as the mean level and change between the two timepoints, were used as outcome variables, predicted by interpersonal stress and internalizing symptoms. All p-values are nominal (uncorrected).*

^1^ Interpersonal Stress was assessed as a latent factor comprised of Negative Parenting (psychological control, level of expressed emotion, and parent-adolescent conflict) and Peer Victimization, repeatedly assessed from age 13 to 17. ^2^ Depressive Symptoms was assessed as a latent factor comprised of Depressive Symptoms repeatedly assessed from age 13 to 17.

**Table S6. Standardized parameter estimates for RQ2.** Structural equation models of DNAm indices of CRP at 17, 25, or mean level and change from age 17 to 25 predicting Depressive Symptoms at age 27.

|  | Ligthart | | | Hillary | | | Wielscher | | |
| --- | --- | --- | --- | --- | --- | --- | --- | --- | --- |
|  | *β* | 95% CI | *p* | *β* | 95% CI | *p* | *β* | 95% CI | *p* |
| Outcome variable: Depressive Symptoms age 27 | DNAm CRP 17 | | | | | | | | |
|  | -.069 | -.185-.046 | .238 | -.045 | -.151-.062 | .411 | -.075 | -.190-.041 | .206 |
|  | DNAm CRP 25 | | | | | | | | |
|  | -.084 | -.207-.038 | .177 | -.102 | -.232-.028 | .124 | -.004 | -.129-.121 | .950 |
|  | Latent Congruence Model | | | | | | | | |
|  | Congruence DNAm CRP 17-25 | | | | | | | | |
|  | -.011 | -.131-.108 | .854 | .023 | -.105-.150 | .729 | -.069 | -.189-.051 | .261 |
|  | Mean level DNAm CRP 17-25 | | | | | | | | |
|  | -.091 | -.206-.025 | .126 | -.078 | -.192-.036 | .182 | -.046 | -.165-.073 | .451 |

*Note. The results of several models are summarized here. Each DNAm CRP score at both 17 and 25, as well as the mean level and change between the two timepoints were used as predictor variables for the outcome depressive symptoms at age 27. All p-values are nominal (uncorrected).*

**Table S7. Standardized parameter estimates for RQ3**. Structural equation mediation models of Interpersonal Stress and Depressive Symptoms from age 13 to 17 predicting age 27 Depressive Symptoms, mediated by DNAm CRP at 17, 25, or mean level or change from 17 to 25.

|  | *β* | 95% CI | *p* | *β* | 95% CI | *p* |
| --- | --- | --- | --- | --- | --- | --- |
|  | DNAm CRP age 17 | | | DNAm CRP age 25 | | |
| Direct effect on Depressive Symptoms age 27 |  |  |  |  |  |  |
| Interpersonal Stress age 13-17^1^ | .026 | -.153-.204 | . 776 | .025 | -.141-.191 | .767 |
| Depressive Symptoms age13-17^1^ | .491 | .312-.669 | .000*** | .494 | .327-.660 | 0.000*** |
| Ligthart | -.076 | -.195-.042 | .207 | -.084 | -.213-.045 | .202 |
| Hillary | -.058 | -.170-.054 | .312 | -.106 | -.239-.027 | .119 |
| Wielscher | -.078 | -.194-.038 | .189 | -.001 | -.131-.129 | .990 |
| Indirect effect on Depressive Symptoms age 27 |  |  |  |  |  |  |
| Interpersonal Stress via Ligthart | -.001 | -.014-.012 | .851 | -.003 | -.021-.016 | .784 |
| Depressive Symptoms via Ligthart | -.007 | -.024-.009 | .379 | -.009 | -.035-.016 | .474 |
| Interpersonal Stress via Hillary | -.006 | -.023-.010 | .436 | .001 | -.028-.030 | .936 |
| Depressive Symptoms via Hillary | -.003 | -.015-.009 | .619 | -.015 | -.048-.017 | .362 |
| Interpersonal Stress via Wielscher | -.002 | -.015-.011 | .767 | .000 | -.006-.006 | .990 |
| Depressive Symptoms via Wielscher | -.002 | -.016-.011 | .749 | .000 | -.004-.004 | .990 |
|  | Latent Congruence Model | | | | | |
|  | Congruence DNAm CRP age 17-25 | | | Mean level DNAm CRP age 17-25 | | |
| Direct effect on Depressive Symptoms age 27 |  |  |  |  |  |  |
| Interpersonal Stress age 13-17^1^ | .026 | -.139-.191 | .754 | “ | “ | “ |
| Depressive Symptoms age 13-17^1^ | .493 | .324-.663 | .000*** | “ | “ | “ |
| Ligthart | -.014 | -.138-.110 | .828 | -.087 | -.208-.034 | .159 |
| Hillary | .020 | -.113-.154 | .764 | - .080 | -.192-.033 | .166 |
| Wielscher | -.072 | -.196-.052 | . 257 | -.043 | -.159-.073 | .471 |
| Indirect effect on Depressive Symptoms age 27 |  |  |  |  |  |  |
| Interpersonal Stress via Ligthart | .000 | -.003-.003 | .950 | -.002 | -.019-.015 | . 826 |
| Depressive Symptoms via Ligthart | .000 | -.003-.003 | .945 | .001 | -.019-.020 | .945 |
| Interpersonal Stress via Hillary | .002 | -.012-.016 | .784 | -.005 | -.022-.012 | .560 |
| Depressive Symptoms via Hillary | -.001 | -.009-.007 | .806 | -.008 | -.032-.016 | .529 |
| Interpersonal Stress via Wielscher | .001 | -.015-.017 | .911 | -.002 | -.011-.008 | .720 |
| Depressive Symptoms via Wielscher | .000 | -.016-.015 | .954 | .001 | -.009 -.010 | .910 |

*Note. The results of several models are summarized here. Each DNAm CRP score at both 17 and 25, as well as the mean level and change between the two timepoints, were used as outcome variables, predicted by interpersonal stress and depressive symptoms. All p-values are nominal (uncorrected).*

Interpersonal Stress was assessed as a latent factor comprised of Negative Parenting (psychological control, level of expressed emotion, and parent-adolescent conflict) and Peer Victimization, repeatedly assessed from TimepointTT1 (age 13) to TimepointT5 (age 17). Similary, Depressive Symptoms were assessed as a latent factor comprised of Depressive Symptoms repeatedly assessed from TimepointTimepointT1 to T5. The effect estimates of the direct effects on Depressive Symptoms at age 27 were only reported once for the model with the Ligthart DNAm scores, since these were very similar across the models including the Wielscher and Hillary scores (e.g. for DNAm 17 .026-.031 for Interpersonal Stress and 484-.491 for Depressive Symptoms)

**Table S8. Standardized parameter estimates for the exploratory models for RQ1.** Structural equation models for Negative Parenting and Peer Victimization separately, predicting DNAm CRP at age 17, age 25, or mean level and change from age 17 to 25.

|  | Ligthart | | | Hillary | | | Wielscher | | |
| --- | --- | --- | --- | --- | --- | --- | --- | --- | --- |
|  | *β* | 95% CI | *p* | *β* | 95% CI | *p* | *β* | 95% CI | *p* |
|  | DNAm CRP age 17 | | | | | | | | |
| Negative Parenting age 13-17^1^ | .106 | -.008-.220 | .067 | .150 | .011-.289 | .035* | .082 | -.047-.210 | .213 |
| Peer Victimization age 13-17^1^ | -.183 | -.341--.026 | .023* | -.070 | -.238-.098 | .413 | -.121 | -.287-.045 | .154 |
| Depressive Symptoms age 13-17^2^ | .177 | .029-.326 | .019* | .100 | -.079-.279 | .275 | .090 | -.080-.260 | .299 |
|  | DNAm CRP age 25 | | | | | | | | |
| Negative Parenting age 13-17^1^ | .129 | -.045-.303 | .146 | -.005 | -.210-.201 | .965 | .063 | -.115-.241 | .488 |
| Peer Victimization age 13-17^1^ | -.139 | -.370-.093 | .240 | -.013 | -.257-.232 | .919 | -.010 | -.233-.212 | .927 |
| Depressive Symptoms age 13-17^2^ | .148 | -.081-.377 | .204 | .159 | -.079-.396 | .190 | .027 | -.188-.242 | .809 |
|  | Latent Congruence Model | | | | | | | | |
|  | Congruence DNAm CRP age 17-25 | | | | | | | | |
| Negative Parenting age 13-17^1^ | -.014 | -.183-.154 | .867 | .134 | -.084-.352 | .228 | .017 | -.164-.198 | .855 |
| Peer Victimization age 13-17^1^ | -.034 | -.245-.178 | .753 | -.053 | -.293-.187 | .665 | -.083 | -.284-.117 | .417 |
| Depressive Symptoms age 13-17^2^ | .049 | -.184-.282 | .680 | -.024 | -.278-.230 | .854 | .061 | -.153-.275 | .579 |
|  | Mean level DNAm CRP age 17-25 | | | | | | | | |
| Negative Parenting age 13-17^1^ | .132 | -.003-.266 | .055 | .082 | -.071-.235 | .293 | .083 | -.059-.225 | .252 |
| Peer Victimization age 13-17^1^ | -.192 | -.378--.007 | .042* | -.046 | -.238-.146 | .640 | -.082 | -.271-.106 | .391 |
| Depressive Symptoms age 13-17^2^ | .186 | .011-.361 | .037* | .143 | -.043-.330 | .132 | .062 | -.118-.243 | .499 |

*Note. The results of several models are summarized here. Each DNAm CRP score at both age 17 and age 25, as well as the mean level and change between the two timepoints, were used as outcome variables, predicted by negative parenting or peer victimization and depressive symptoms. All p-values are nominal (uncorrected).*

^1^ Interpersonal Stress was assessed as a latent factor comprised of Negative Parenting (psychological control, level of expressed emotion, and parent-adolescent conflict) and Peer Victimization, repeatedly assessed from age 13 to 17.

^2^ Depressive Symptoms was assessed as a latent factor comprised of Depressive Symptoms repeatedly assessed from age 13 to 17.

**Table S9. Standardized parameter estimates for the exploratory models for RQ3.** Structural equation models of Negative Parenting and Peer Victimization separately, as well as Depressive Symptoms from age 13 to 17, predicting age 27 Depressive Symptoms, mediated by DNAm CRP at age 17, age 25, or mean level and change from 17 to 25.

|  | *β* | 95% CI | *p* | *β* | 95% CI | *p* |
| --- | --- | --- | --- | --- | --- | --- |
|  | DNAm CRP age 17 | | | DNAm CRP age 25 | | |
| Direct effect on Depressive Symptoms age 27 |  |  |  |  |  |  |
| Negative Parenting age 13-17^1^ | .041 | -.099-.180 | .570 | .038 | -.106-.183 | .603 |
| Peer Victimization age 13-17^1^ | -.050 | -.228 -.127 | .577 | -.047 | -.230-.136 | .617 |
| Depressive Symptoms age 13-17^1^ | .519 | .330-.708 | .000*** | .517 | .320-.714 | .000*** |
| Ligthart | -.086 | -.207-.034 | .160 | -.080 | -.216-.055 | .244 |
| Hillary | -.062 | -.170-.045 | .256 | -.108 | -.243-.028 | .121 |
| Wielscher | -.086 | -.201-.029 | .143 | .007 | -.124-.138 | .911 |
| Indirect effect on Depressive Symptoms age 27 |  |  |  |  |  |  |
| Negative Parenting via Ligthart | -.009 | -.025-.007 | .253 | -.010 | -.031-.011 | .348 |
| Peer Victimization via Ligthart | .016 | -.010-.041 | .227 | .011 | -.017-.040 | .434 |
| Depressive Symptoms via Ligthart | -.015 | -.041-.011 | .262 | -.012 | -.043-.019 | .449 |
| Negative Parenting via Hillary | -.009 | -.028-.010 | .334 | .001 | -.022-.024 | .911 |
| Peer Victimization via Hillary | .004 | -.010-.019 | .541 | .002 | -.026-.030 | .892 |
| Depressive Symptoms via Hillary | -.006 | -.024-.011 | .467 | -.017 | -.055-.021 | .376 |
| Negative Parenting via Wielscher | -.007 | -.022-.007 | .336 | .000 | -.008-.009 | .913 |
| Peer Victimization via Wielscher | .010 | -.010-.030 | .318 | -.000 | -.002-.002 | .937 |
| Depressive Symptoms via Wielscher | -.007 | -.027-.012 | .451 | .000 | -.003-.004 | .913 |
|  | Latent Congruence Model | | | | | |
|  | Congruence DNAm CRP age 17-25 | | | Mean level DNAm CRP age 17-25 | | |
| Direct effect on Depressive Symptoms age 27 |  |  |  |  |  |  |
| Negative Parenting age13-17^1^ | .042 | -.098-.181 | .557 | “ | “ | “ |
| Peer Victimization age 13-17^1^ | -.052 | -.233-.130 | .576 | “ | “ | “ |
| Depressive Symptoms age 13-17^1^ | .521 | .331-.712 | .000*** | “ | “ | “ |
| Ligthart | -.025 | -.151-.100 | .692 | -.091 | -.210-.028 | .134 |
| Hillary | .019 | -.114-.151 | .784 | -.084 | -.200-.032 | .157 |
| Wielscher | -.085 | -.207-.037 | .170 | -.043 | -.164-.079 | .491 |
| Indirect effect on Depressive Symptoms age 27 |  |  |  |  |  |  |
| Negative Parenting via Ligthart | .000 | -.004-.005 | .903 | -.012 | -.031-.007 | .228 |
| Peer Victimization via Ligthart | .001 | -.007-.008 | .831 | .017 | -.011-.046 | .233 |
| Depressive Symptoms via Ligthart | -.001 | -.011-.008 | .799 | -.016 | -.045-.012 | .262 |
| Negative Parenting via Hillary | .003 | -.016-.021 | .790 | -.007 | -.023-.009 | .417 |
| Peer Victimization via Hillary | -.001 | -.009-.007 | .809 | .004 | -.014-.022 | .662 |
| Depressive Symptoms via Hillary | -.000 | -.006-.005 | .892 | -.012 | -.037-.013 | .349 |
| Negative Parenting via Wielscher | -.001 | -.017-.015 | .871 | -.004 | -.015-.008 | .546 |
| Peer Victimization via Wielscher | .007 | -.015-.030 | .532 | .003 | -.010-.017 | .613 |
| Depressive Symptoms via Wielscher | -.005 | -.027-.017 | .649 | -.002 | -.014-.009 | .674 |

*Note. The results of several models are summarized here. Each DNAm CRP score at both age 17 and age 25, as well as the mean level and change between the two timepoints, were used as outcome variables, predicted by interpersonal stress and depressive symptoms.*

^1^ Interpersonal Stress was assessed as a latent factor comprised of Negative Parenting (psychological control, level of expressed emotion, and parent-adolescent conflict) and Peer Victimization, repeatedly assessed from age 13 to 17. Depressive Symptoms was assessed as a latent factor comprised of Depressive Symptoms repeatedly assessed from age 13 to 17. The effect estimates of the direct effects on Depressive Symptoms at age 27 were only reported once for the model with the Ligthart DNAm scores, since these were very similar across the models including the Wielscher and Hillary scores (e.g. for DNAm 17 all .041 for Negative Parenting, -0.052--0.041 for Peer Victimization and .510-.519 for Depressive Symptoms).

**Table S10. Intraclass correlation (ICC) of repeated measurements of DNAm indices of CRP.**

| DNAm index of CRP | ICC age 17 and age 25 | p-value |
| --- | --- | --- |
| Ligthart | .57 | 1.6e-18 |
| Hillary | .31 | 6.7e-05 |
| Wielscher | .30 | 8.7e-05 |


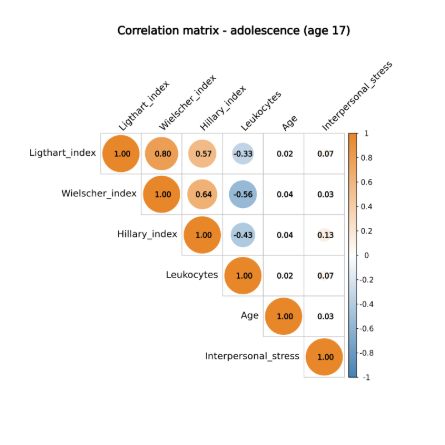

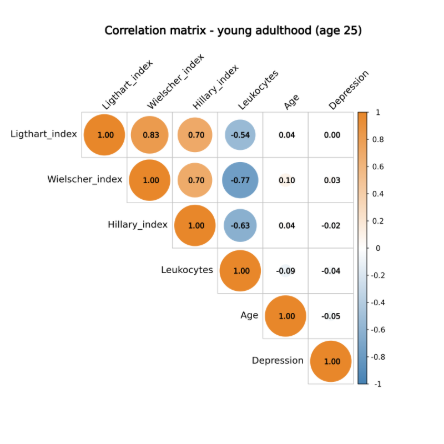


**Figure S11. Covariance plots for the DNAm-estimated proportion of leukocytes in saliva with DNAm indices of CRP, age, interpersonal stress, and depression.** DNAm sampling performed ~age 17 and ~age 25. We estimated the fraction of leukocytes versus epithelial cells from DNAm data using the saliva reference panel for studies in children by Middleton et al. (2020) as implemented in the ewastools package. Since leukocytes and epithelial cell fractions add up to 1 and showed near-perfect correlation (-0.99), we only include the leukocyte fraction to avoid redundancy. The interpersonal stress variable in adolescence refers to the latent factor score informed by 5 annual measurement waves from age 13-17 of both negative parenting and peer victimization. The depression variable in young adulthood refers to self-reported depressive symptoms at age 26, the main outcome in our study.
